# Forming-less and Non-Volatile Resistive Switching in WO_X_ by Oxygen Vacancy Control at Interfaces

**DOI:** 10.1038/s41598-017-10851-8

**Published:** 2017-08-31

**Authors:** Seokjae Won, Sang Yeon Lee, Jucheol Park, Hyungtak Seo

**Affiliations:** 1 Department of Energy Systems Research, Ajou University, Suwon, 16499 Republic of Korea; 2 Gyeongbuk Science Technology Promotion Center, Gumi Electronics & Information Technology Research Institute, Gumi, 39717 Gyeongbuk Republic of Korea; 3 Department of Materials Science and Engineering, Ajou University, Suwon, 16499 Republic of Korea

## Abstract

Resistive switching devices are recognized as candidates for next-generation memory devices in that they can replace conventional memory devices. In these devices, a WO_X_ film deposited by RF magnetron sputtering with a significant number of oxygen vacancies exhibits a resistive switching property and does not involve the use of a forming process. The resistive switching mechanism involves the hopping of electrons through the sub-band states of the oxygen vacancies in E-field-driven electromigration. X-ray photoemission spectroscopy, ultra-violet photoemission spectroscopy, and transmission electron microscopy-electron energy loss spectroscopy were performed to analyze local variations in the O-vacancies and in the electronic band structure of a WO_X_ thin film. The band structure is responsible for the correlation between the motion of the electrons under the interface effect at the electrodes with the change in the resistance and the bias-polarity dependence of the I-V property of the device. The optimized metal-insulator-metal structure (Pt/WO_X_/Au), which has an asymmetric electrode and many oxygen vacancies, gives rise to excellent resistive-switching properties with a high on/off ratio on the order of 10^5^ times, a low set voltage of <0.34 V, and a uniform DC cyclic performance in the order of 1500 cycles at room temperature. These specifications can be further adopted for application to non-volatile memory-device applications.

## Introduction

In spite of the ongoing reduction in the dimensions of conventional Si-based flash-memory devices, it is expected that there will come a point where physical limitations will prevent any further reduction in device size^1–5^. Many researchers have been searching for a promising alternative to non-volatile memory to overcome this issue. Resistive switching behavior has emerged as a strong candidate for the next generation of non-volatile memory given its structural simplicity, high scalability, low power consumption, fast switching speed, and high-density integration^6–9^. A key aspect of resistive random access memory (ReRAM) is the use of the switching of the resistance mode of the insulator. Therefore, many studies have reported on insulators with a resistive-switching behavior, whereby the insulator can reversibly switch between a high-resistance state and a low-resistance state^10^. This resistive switching behavior can be explained by two mechanism models; filamentary and non-filamentary switching^8^. Filamentary resistive random access memory (ReRAM) features a forming process whereby filaments are formed to link the bottom electrode to the top electrode^10^. While the conductive filaments are aligned, the resistance is low. Then, the rupturing of these filaments, as a result of electrochemical redox and Joule heating, makes the resistance high^8^. On the other hand, it is generally agreed that the mechanism responsible for the behavior of non-filamentary ReRAM is resistive switching caused by the migration of oxygen vacancies during the application of a voltage. This plays a crucial role in the resistive switching behavior^8, 10^. Unipolar or bipolar resistive switching behaviors have been identified in various types of materials such as perovskite, transition metal oxide (TMO), chalcogenide, and organics^1, 3, 4, 8, 11–14^. Among them, TMO has a particularly large number of candidates; NiO, TiO_2_, TaO_2_, HfO_2_, WO_3_ and (Ba,Sr)TiO_3_^1, 15–19^. Notably, WO_3_ has been identified as being well suited for application as an active layer in ReRAM due to its excellent compatibility and simple process^13^. Although some reports focusing on ReRAM device performance have been published^13, 20, 21^, in-depth analyses of material tuning and the switching mechanism of WO_3_ have been relatively lacking. It has been reported that, in the case of WO_3_, it is important to have a wide band gap with a resistive switching behavior^8^, although the behaviors and distribution of the oxygen vacancies (O-vacancy) in the WO_3_ are unknown in spite of its importance to non-filamentary resistive switching. Since WO_3_ is known to be a highly reactive material due the large quantity and chemical reversibility of O-vacancies, the investigation of this property of WO_3_ would be invaluable to the optimization of TMO-based ReRAM. Herein, we demonstrate an improved approach to the fabrication of WO_X_ ReRAM with non-filamentary resistive switching, based on the migration of oxygen vacancies. A non-crystalline WO_X_ thin film was deposited using RF magnetron sputtering at a low temperature (near room temperature, 40 °C). WO_X_ conforms to the depletion model for ReRAM operation, whereby control depends on there being more oxygen vacancies than WO_3_, and where the oxygen vacancies can be easily redistributed to form a path for a charge. Considerably reliable ReRAM, with a of Pt/WO_X_/Au metal-insulator-metal (MIM) structure, exhibits outstanding ReRAM electrical properties such as a high on/off ratio in the order of 10^5^ times, a low set voltage of less than 0.34 V, and a uniform DC cyclic performance of around 1500 cycles at room temperature, even if deposited at near room temperature. The results of the present study suggest the pivotal role of oxygen vacancies and the relevant energy band structure. These were experimentally probed by the application of spectroscopic techniques, to explain the mechanism of resistive switching and the characteristics of the I-V curve. Furthermore, we proved the role of electrodes, which are used in MIM structure to make the effect of rectifying junction.

## Results

### Scheme and electrical properties of resistive switching

**Figure 1:**
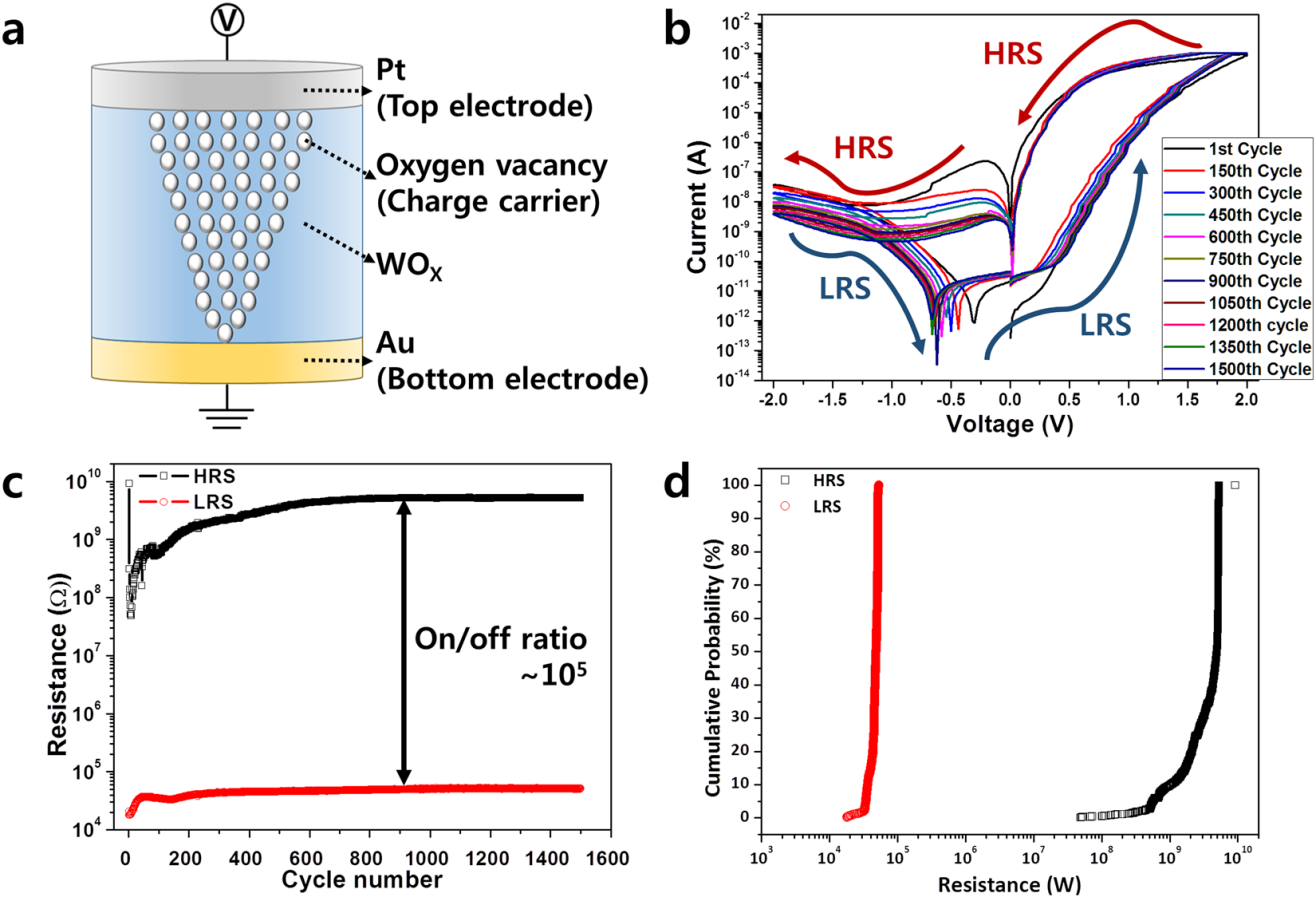
Scheme and I–V characteristics; (**a**) Schematic of Pt/WO_X_/Au structure of resistive switching mechanism with oxygen vacancies, (**b**) I–V curves for 1500 cycles of DC voltage from 2.0 to −2.0 V with counter-clockwise bias sweep, (**c**) Endurance for 1500-cycle DC-bias and resistance between HRS and LRS, (**d**) Cumulative probabilities of MIM structure for 1500 cycles.

Figure 1a shows the general scheme of the resistive switching in a WO_x_-based metal (Pt)-insulator-metal (Au) ReRAM with oxygen vacancy distribution. The white circles represent the oxygen vacancies which act as the conductive channel for charges. When Pt, configured as the top electrode, is positively biased, oxygen vacancies migrate and then accumulate to form a path, allowing many electrons to pass through the insulator (WO_X_) between the top electrode (Pt) and bottom electrode (Au). When Au, configured as the top electrode, is positively biased, the Joule heating destroys the oxygen vacancies, leading to the rupture of the conductive channel^22, 23^. Figure 1b shows the typical progression of resistive switching at room temperature. A voltage is applied in the sequence of 0.0 V → 2.0 V → 0.0 V → −2.0 V → 0.0 V at a biasing interval of 0.02 V. Then, the current exhibits counterclockwise switching (CCWS). When the voltage bias on the top electrode is positive, the resistance changes from the high state to the low state without any extra process such as the forming needed to make a metallic filament. This change in the resistance allows a greater current to flow. As shown indicated by the current-voltage (I-V) curve, however, the current does not increase instantly and steeply but instead increases gradually. This switching property is different from that of a metallic filament^10, 24^. The set voltages are less than 0.5 V and are about 0.34 V on average. I-V curves with uniform and low set/reset voltages are shown for up to 1500 DC sweeps. This large number of DC cycles is comparable to that of previously reported WO_X_ ReRAM^1, 8, 13, 18, 25–27^. It can be seen that the current which flows when a negative voltage is applied is suppressed but when a positive voltage is applied, a high current flows, in the same way as in a rectification diode. The DC endurance characteristics between the high-resistance state (HRS) and low-resistance state (LRS) at 0.34 V are shown in Fig. 1c. The on/off ratio between the HRS and LRS is up to 10^5^ times and remains stable for 1500 cycles or more. While the LRS remains stable for 1500 cycles, the HRS is slightly unstable in the early stages but becomes more stable after approximately 550 cycles. The cumulative probability for 1500 cycles is shown in Fig. 1d. As expected, the Pt/WO_X_/Au exhibits a resistive switching behavior with a good distribution of the LRS and a slightly unstable distribution of the HRS.

### Control groups

**Figure 2:**
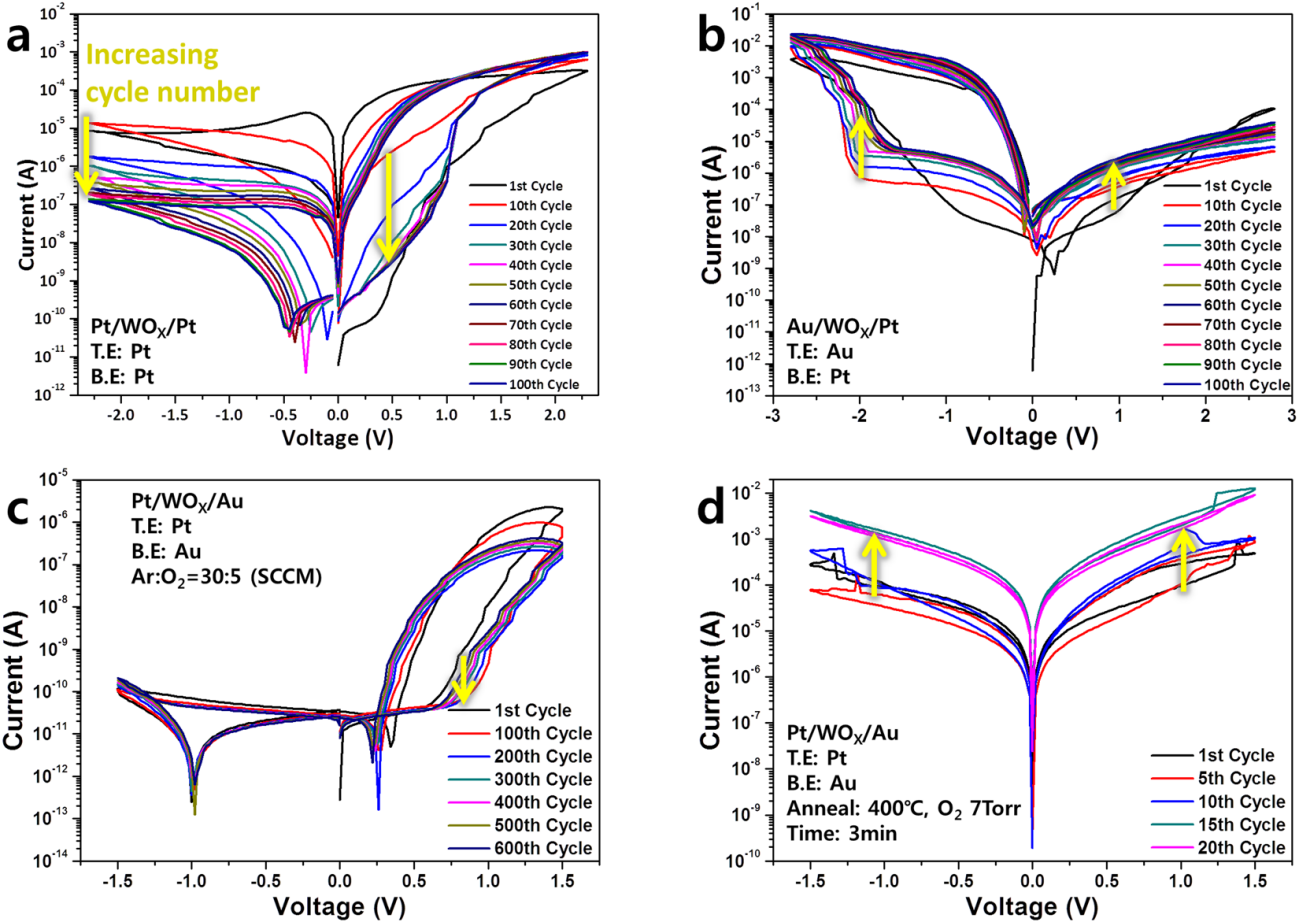
I–V curves of control devices for comparison; (**a**) Pt/WO_X_/Pt device for 100 cycles from 2.5 to −2.5 V, (**b**) Au/WO_X_/Pt device for 100 cycles from 3.0 V to −3.0 V, (**c**) Pt/WO_X_/Au deposited by RF magnetron sputtering in oxygen-rich atmosphere for 600 cycles between 1.5 and −1.5 V, (**d**) Pt/WO_X_/Au annealed at 400 °C and 7 Torr in an oxygen atmosphere for 20 cycles between 1.5 V and −1.5 V.

**Figure 3:**
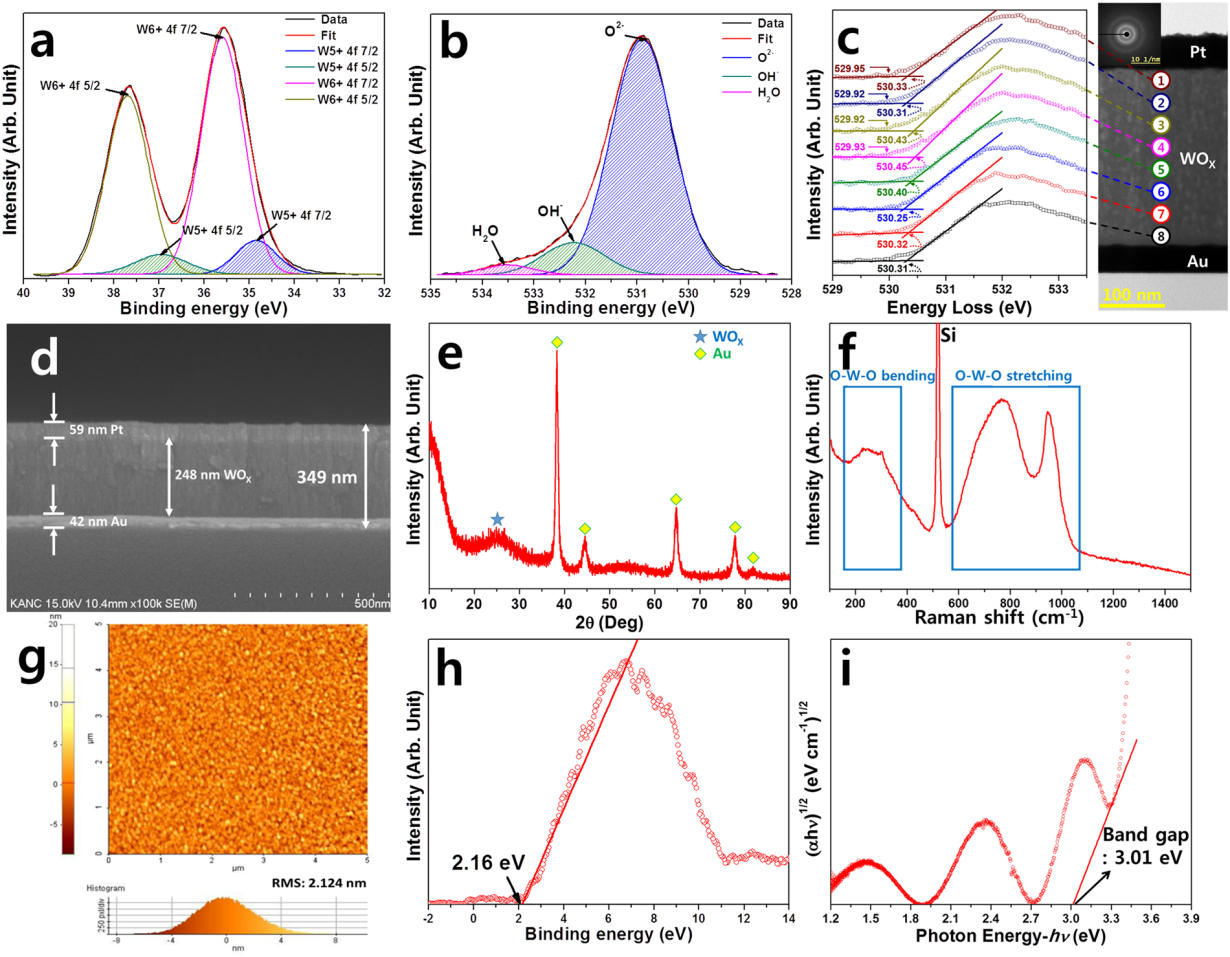
Chemical and structural analysis of WO_X_; (**a**) XPS narrow scan of W 4*f* at the surface which was peak-deconvoluted with sub-peaks for each chemical binding state, (**b**) O 1*s* XPS of WO_X_, (**c**) cross-sectional TEM image and depth-wise EELS analysis which indicates OK1 edge spectra with onset energies (solid arrows) and secondary onset energies (dotted arrows), (**d**) SEM image of Pt/WO_X_/Au device, (**e**) XRD data of amorphous WO_X_ on Au substrate, (**f**) Raman data of amorphous WO_X_ measured using 532-nm laser excitation, (**g**) AFM image of surface of WO_X_ device, (**h**) XPS valence band maximum of WO_X_ device as determined by spectrum onset binding energy, and (**i**) band gap determination of WO_X_ device throughout UV-vis measurement; the oscillation in the spectra from 1.2 to 3 eV is due to the internal optical interference pattern. The intrinsic absorption starts from around 3 eV near the band gap energy of WO_3_.

**Figure 4:**
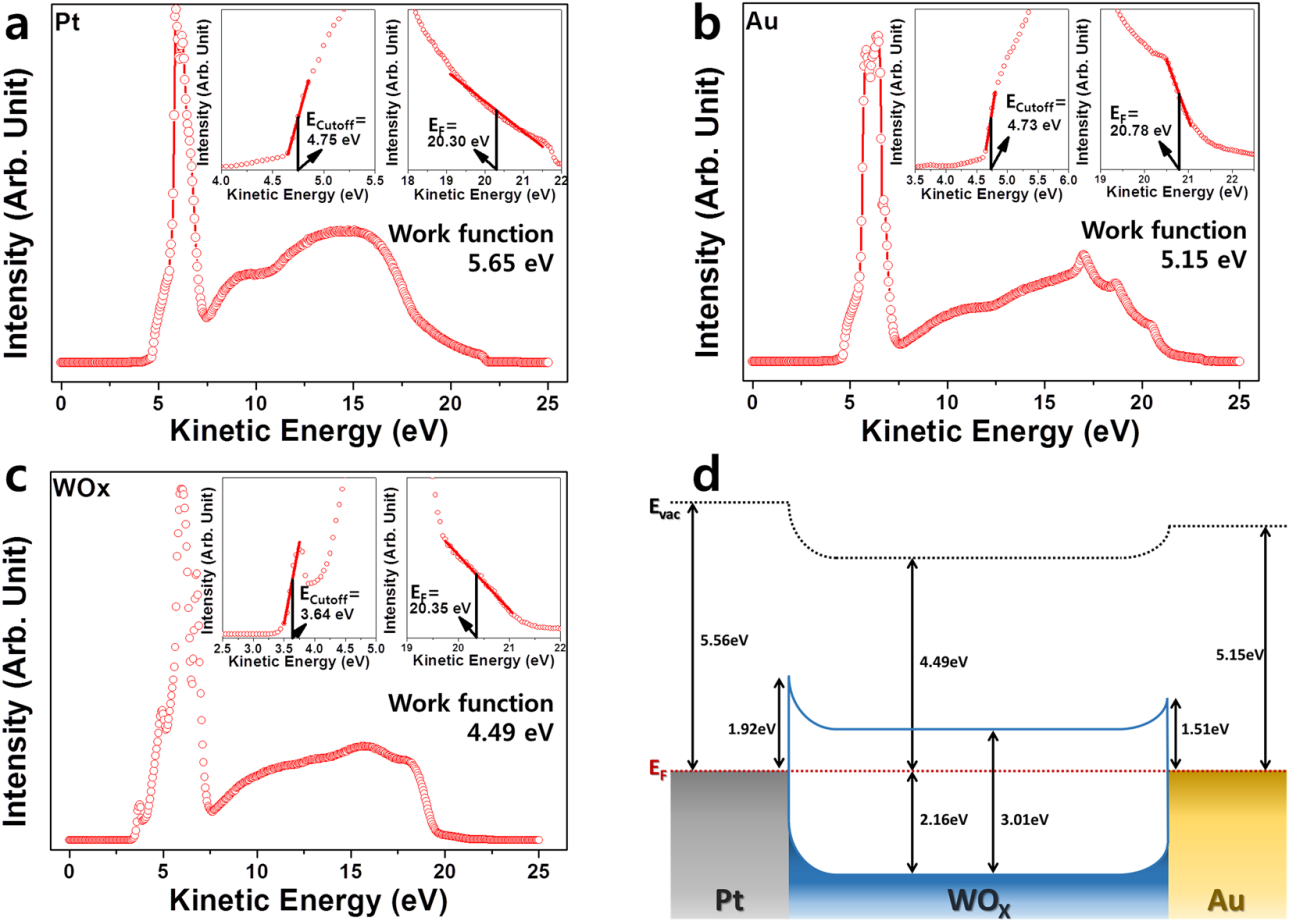
UPS analysis for the work function extraction and construction of band structure to explain the mechanism; UPS spectra of (**a**) Pt, (**b**) Au, and (**c**) WO_X_ with the work functions of the top and bottom electrodes, respectively. The inset figures indicate the extraction of E_cutoff_ and E_F_ energy from the high- and low-energy shoulders in the UPS spectra. (**d**) Band structure of WO_X_ device without voltage (at thermal equilibrium).

To identify the physical origins of the electrical properties of Pt/WO_X_/Au, various types of control devices were fabricated. Figure 2 shows the effect of the electrodes and oxygen vacancies in WO_x_. A Pt/WO_X_/Pt device is shown in Fig. 2a. It has the same Pt electrodes at both the top and bottom. The overall I-V curve feature of the Pt/WO_X_/Pt device differs from that of the Pt/WO_X_/Au device, and the rectification under bias is much weaker than that of the reference (Pt/WO_X_/Au) device. The cyclic performance and on/off ratio were also worse and the set/reset voltage was much higher than those of the reference device. There are the rectification properties in I-V curve, because oxygen vacancies are concentrated at top of device near top electrode (this will be more discussed in Fig. 3c.). In addition, Fig. 2b shows the characteristics of the device with the electrodes switched between the top and bottom, that is, Au/WO_X_/Pt. The resistive switching characteristic is opposite to that of the reference group. In the reference group, the marked resistive switching characteristic appears when the positive voltage is applied. However, the control device shown in Fig. 2b shows the marked resistive switching characteristic at the negative voltage. Therefore, Fig. 2a and b confirm the roles of the two kinds of electrodes on the bias-polarity asymmetric switching behaviors, rectification, and on/off ratios. Rectification properties are confirmed in Fig. 2a and b due to differences of electrode work function and position of oxygen vacancies (this will be further discussed in Fig. 4.). The two control devices shown in Fig. 2c and d were fabricated to confirm the role of the oxygen vacancies in WO_x_. The WO_x_ deposition shown in Fig. 2c was carried out in an oxygen-rich atmosphere (Ar:O_2  _ = 30:5 SCCM). This amount of oxygen was 10 times greater than the standard amount used for the reference device. The control device shown in Fig. 2c has a much lower on/off ratio and higher set voltage than the reference device. The set voltage is 0.62 V at the 600^th^ cycle, at which the on/off ratio is 617. The device with higher oxygen concentration requires a higher set voltage, while the gap between the HRS and LRS decreases. Figure 2d shows the I-V curves for a device deposited under the same Ar/O_2_ conditions (Ar:O_2  _ = 30:5 SCCM) as those used for the reference group but subsequently annealed at 400 °C, under an O_2_ pressure of 7 Torr for 3 min. The annealed device does not exhibit any resistive switching property at all and its base current is too high, even though this device has a lack of oxygen vacancies due to the oxygen annealing. This result may be due to the increase in the grain boundaries because of the thermal crystallization of the WO_x_ film, which can be a conductive path. It was experimentally confirmed, from Fig. 2, that the electrodes and oxygen vacancies in the WO_x_ greatly affect the resistive switching in the control devices.

### XPS and TEM-EELS analysis

To characterize the binding state and valence band electronic structure of WO_x_, an XPS analysis was performed. The W 4 f spectra shown in Fig. 3a were deconvoluted into four doublets by Gaussian fitting, corresponding to W^5+^ 4*f*_5/2_ (36.93 eV), W^5+^ 4*f*_7/2_ (34.83 eV), W^6+^ 4*f*_5/2_ (37.68 eV), and W^6+^ 4*f*_7/2_ (35.58 eV)^28–31^. Although the deposition process was carried out using the WO_3_ target, the XPS data indicates that the tungsten oxide thin film is either not fully oxidized (*i.e.,* WO_X_, X < 3) or incorporates oxygen vacancies. The peak area ratio of W^5+^/W^6+^ is 6.38%, which indicates a considerable O deficiency in the oxide. Figure 3b shows the O 1*s* spectrum for the WO_X_ thin film. This peak was also deconvoluted into three components, namely, O^2−^, OH^−^, and H_2_O. The binding energy of O^2−^ is about 530.88 eV, corresponding to the strong W=O bonds^32^. The binding energy of OH^−^ is about 532.23 eV and the binding energy of H_2_O is about 533.53 eV. Table 1 lists the binding energies and atomic composition ratios. The stoichiometric ratio between tungsten and oxygen can be inferred from the composition ratio. The atomic ratio of tungsten is 17.95% and the atomic ratio of oxygen is 49.48%. Therefore, the ratio of tungsten to oxygen is about 1:2.76 (WO_2.76_), not 1:3 (WO_3_). This indicates that this WO_X_ contains a large number of oxygen vacancies. In the cross-sectional scanning TEM-EELS analysis, the selected area electron diffraction (SAED) pattern does not show any pattern indicating a degree of crystallinity but merely indexes the ambiguous diffraction patterns shown in Fig. 3c, that is, the amorphous phase. Figure 3d shows a cross-sectional SEM image of the Pt/WO_X_/Au MIM structure, which consists of uniformly staked structures. The WO_X_ film exhibits a columnar structure with an amorphous phase. This amorphous WO_X_ phase can be confirmed from the XRD data shown in Fig. 3e, which exhibits a broad peak at approximately 25°. In addition, the Raman results clearly show the presence of a WO_x_ film consisting of O-W-O bending and the stretching mode in Fig. 3f ^33^. The surface roughness of the WO_X_ was estimated to have a root-mean-square (RMS) roughness of 2.124 nm of by AFM analysis (Fig. 3g). The uniform WO_X_ surface is crucial because its interface with the electrode has a major influence of the electrical properties, such as the on/off ratio, switching uniformity, and cyclic performance. An RMS roughness of 2.124 nm is reasonable in terms of deposition by RF magnetron sputtering. This is because the deposition was carried out at around room temperature with no driving force for the crystallization. Figure 3c shows how EELS analysis was performed to confirm the depth-wise distribution of oxygen vacancies, relative to the amount of oxygen vacancies at the surface, as determined by XPS. The EELS results show an O K1 excitation from 529 eV to 533.5 eV. The evolution of the O K1 onset energy is fitted, with the dotted arrows indicated the plotted onset energy points. On the other hand, the solid arrows indicate those points at which the secondary onset energy starts (applied to positions 1 to 4). The dotted arrows correspond to strongly bound oxygen, while the solid arrows indicate less strongly bound oxygen. The presence of weak bonds means that there is an oxygen vacancy, but not a full oxidation state. It can be seen that the secondary onset energies are clear, with those in the upper region (near the top electrode) being much more distinctive. As a result, EELS shows that there are more oxygen vacancies in the upper region near the top Pt electrode. Figure 3h and i identify a valence band maximum at 2.16 eV, based on the Fermi energy (E_F_) level as determined by XPS analysis and an optical bandgap at 3.01 eV, as apparent in the Tauc plot of the absorption spectrum of the WO_X_ film. These VBM and bandgap values indicate that the WO_X_ film is an n-type semiconductor. The oscillating pattern of the Tauc plot below 3 eV, shown in Fig. 3i, is due to the internal optical interference inside the WO_X_.

**Table 1:** Summary of atomic ratios of W, O, C, and N, as calculated from XPS peak areas of W 4*f*, O 1*s*, C 1*s*, and N 1*s* by considering the sensitivity factor of each element.

Material	Binding Energy (eV)	Atomic ratio (%)
W 4*f*	35.54	17.95
O 1*s*	527.86	49.48
C 1*s*	284.55	29.60
N 1*s*	402.24	2.97

### UPS and band alignment

UPS and XPS analyses were performed to construct the band structure and explain the behavior of the carrier transport and injection. Figure 3h shows the valence band maximum as determined from the XPS analysis, which points to an energy gap at 2.16 eV between valence band maximum and Fermi energy level. It was previously reported that a partially filled W 5*d* state appears below 3 eV in the VB edge XPS spectra^34^. Furthermore, the UV-vis data exhibits a band gap of WO_X_, as shown in Fig. 3i. There are some oscillating spectra between 1.2 eV and 3.0 eV due to the internal optical interference and the intrinsic absorption from around 3.0 eV near the band gap energy of WO_3_. Therefore, we can say that the WO_X_ band gap is 3.01 eV, as determined from the UV-vis observations.

Figure 4a and b show UPS data indicating the work functions of the Pt and Au used as an electrode. The following equation expresses the photoelectron emission of UPS:

1 $${E}_{kin}=h\omega -\phi -{E}_{b},$$

where E_kin_ is the electron kinetic energy, hω is the photo energy of light (He UV emission at 21.22 eV), φ is the work function of the specimen, and E_b_ is the binding energy. Therefore, the equation can be revised to calculate the work function from the UPS data:

2 $${\rm{\phi }}=h{\rm{\omega }}-|{E}_{cutoff}-{E}_{F}|,$$

where E_cutoff_ and E_F_ can be represented by fitting the energy graph of the UPS at high and low kinetic energies. Using these equations, it was confirmed that the work functions of the top Pt electrode and the bottom Au electrode were 5.56 and 5.15 eV, respectively. In Fig. 4c, the UPS data for the WO_X_ is shown. The work function of the WO_X_ was observed to be 4.49 eV. The band structure based on the measured UPS, XPS, and UV-vis data is shown in Fig. 4d. Schottky barriers occur at the Pt/WO_X_ and WO_X_/Au interfaces, due to the differences in the Fermi energy level. The Schottky barrier at the interface with the top electrode is much larger than that at the bottom electrode due to the difference in the work function. This suggests that the metal/WO_X_ interfaces have a major impact on electron injection. Especially, the asymmetric Schottky barrier at the two interfaces may have a greater influence on the behavior of the electrons, depending on the polarity of the voltage applied to the device.

## Discussion

**Figure 5:**
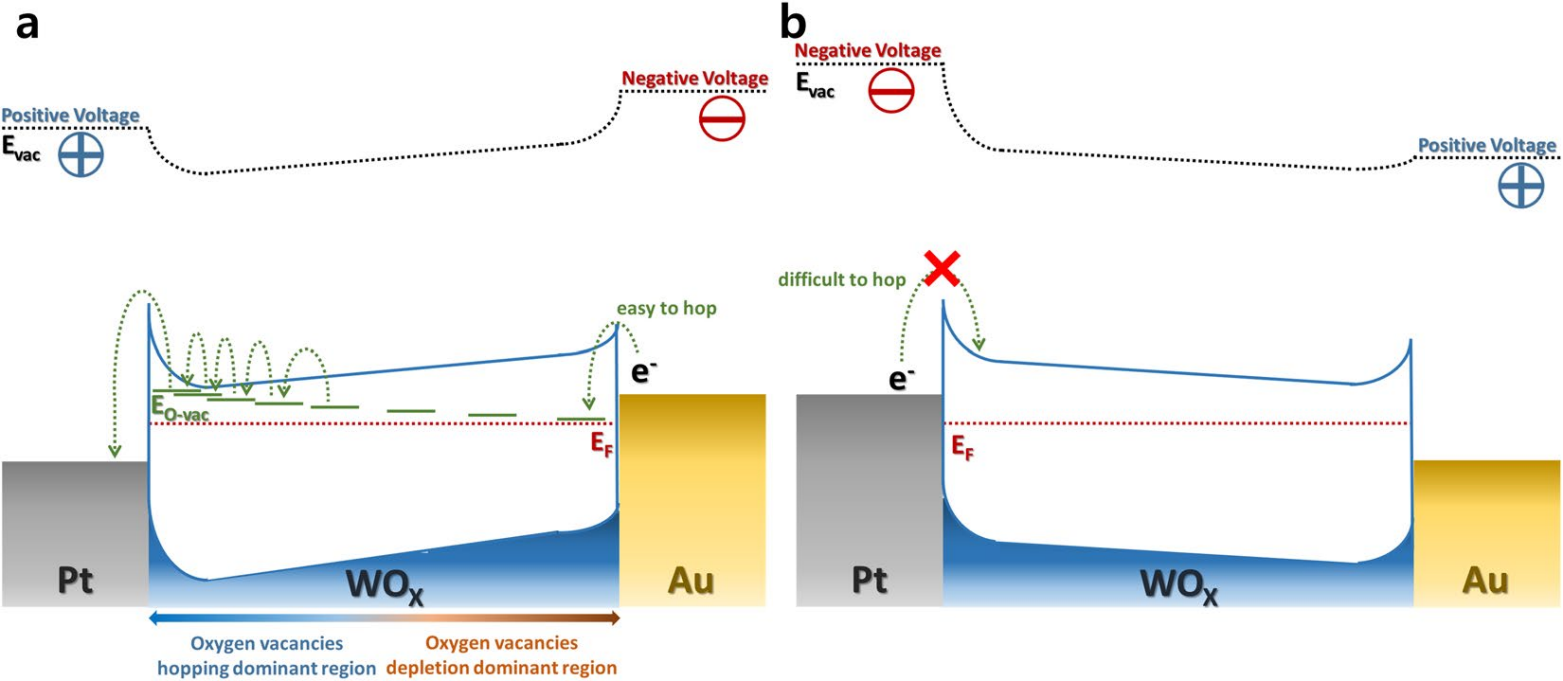
Band structures under biasing condition; Band structure showing (**a**) electron hopping from bottom (Au) to top (Pt) electrode *via* sub-bands of oxygen vacancies when the positive voltage is applied to the top electrode. As the concentration of oxygen vacancies increases, sub-bands become shallower states and (**b**) difficulty in hopping of electron from top to bottom electrode when a negative voltage is applied to the top electrode.

When a voltage is applied, the band structure is changed due to the formation of a quasi Fermi energy level. Figure 5a shows that, when a positive voltage is applied to the top electrode, a corresponding negative voltage is applied to the bottom electrode. When a negative voltage is applied to the bottom electrode, the Schottky barrier is lowered and electrons are easily injected into the top electrode. However, as shown by the I-V curve, the current does not increase immediately but increases gradually and step-wise, pointing to the double process of carrier injection and hopping. The oxygen vacancies form partially electron-filled W 5*d* and form sub-band edge states where the electrons can hop^34^. Because the Schottky barrier at the interface near the bottom electrode is low, electrons can readily pass through at the interface and then the electrons can move *via* the sub-band edge states of oxygen vacancies^16, 35–38^. Thus, as a result of this process, the current increases stepwise. Figure 5b shows the band structure when a negative voltage is applied to the top electrode. The Schottky barrier at the interface near the top electrode is too high for electrons to pass easily. This may be the reason why the I-V curves are similar to those of rectification junctions. The band alignments also can explain the shape of the I-V curves in control devices. The current flows consist of carrier injection at the Schottky interface in one direction only due to the difference in the work functions of the electrode and carrier transport as a result of the oxygen vacancies in the WO_X_, a key element of the resistive switching. Therefore, the resistive switching devices in the control devices exhibit poor properties, suggesting the importance of the optimization of the interface and resistive oxide stoichiometry for improved ReRAM performance.

## Conclusion

We successfully fabricated a WO_X_ film based on ReRAM devices using RF magnetron sputtering with a WO_3_ target. The combined spectroscopic analysis using XPS, the TEM-SAED pattern, and STEM-EELS results revealed the actual distribution of the oxygen vacancies in the WO_X_ film and its top and bottom interfaces with the metal electrodes. Oxygen vacancies inside the WO_X_ film form a sub-band edge, which helps the electrons to move through the WO_X_ channel while the voltage-driven electromigration of the oxygen vacancies is responsible for the resistance between the HRS and LRS. The rectifying I–V curve is also explained by the asymmetric interfacial alignments (i.e., the different Schottky barrier energies) due to the work function difference between the Pt and Au electrodes, as confirmed by UPS. The optimized resistive switching Pt-WO_2.76_-Au MIM device has a stable DC cyclic property at 1500 cycles and outstanding on/off ratio of about 10^5^ times. Therefore, this strategy demonstrates an effective means of fabricating the WO_X_-based resistive switching device using RF magnetron sputtering at around room temperature.

## Experimental

To form the bottom electrode, Au with a thickness of approximate 50 nm was deposited on an SiO_2_ wafer using an E-beam evaporator. The resistive switching channel for formed by depositing WO_X_ with a thickness of about 200 nm at 40 °C using RF magnetron sputtering. As the sputter source material, WO_3_ (purity: 99.99%) with a diameter of 2 inches was used. The base pressure before the injection of reacting gas was lower than 4.0 × 10^−7^ Torr. As the reacting gas, argon and oxygen were introduced into the sputter chamber at flow rates of 30 and 0.5 SCCM, respectively, to maintain a working pressure of 20 mTorr for the film deposition. The deposition was carried out at an RF power of 150 W for 30 min. For the top electrode, Pt was deposited to a thickness of 50 nm by E-beam evaporation using a mask with a diameter of 50 μm. A metal-insulator-metal (MIM) structure was fabricated through this process to evaluate the ReRAM characteristics. X-ray diffraction (XRD, Rigaku MiniFlex-II Desktop) and high-resolution Raman spectroscopy (Raman, HORIBA Jobin Yvon LabRam HR Evolution) were used to observe the element and crystal structure of the thin film. The surface of the thin film was confirmed using an atomic force microscope (AFM, Tecsco Multi-mode SPM). A field-emission scanning electron microscope (FESEM, Hitachi S-4800) was used to check the thickness of the top and bottom electrodes and the insulator. The I–V characteristics of the electrical properties were measured using a probe station (Keithley 4200SCS). X-ray photoelectron spectroscopy (XPS, Thermo Fisher Scientific Co., theta probe base system) was used to identify the chemical compositions of the WO_X_ thin film at a binding energy resolution of 0.05 eV. The XPS binding energy calibration was set to the reference C-C bonding (284.55 eV) in C1s. To check the local crystalline structure and the depth-resolved composition changes, Cs-corrected field emission transmission electron microscopy (TEM, JEM-ARM 200 F) was used. A specimen using a focused ion beam (FIB, NOVA 600 Nanolab (FEI)) was used for the cross-sectional TEM analysis.
